# Characterization of a Bioflocculant (MBF-UFH) Produced by *Bacillus* sp. AEMREG7

**DOI:** 10.3390/ijms160612986

**Published:** 2015-06-08

**Authors:** Kunle Okaiyeto, Uchechukwu U. Nwodo, Leonard V. Mabinya, Arinze S. Okoli, Anthony I. Okoh

**Affiliations:** 1 South Africa-Medical Research Council (SA-MRC), Microbial Water Quality Monitoring Centre, University of Fort Hare, 5700 Alice, South Africa; E-Mails: unwodo@ufh.ac.za (U.U.N.); lmabinya@ufh.ac.za (L.V.M.); aokoh@ufh.ac.za (A.I.O.); 2 Applied and Environmental Microbiology Research Group, Department of Biochemistry and Microbiology, University of Fort Hare, 5700 Alice, South Africa; 3 GenØK-Centre for Biosafety, Forskningsparken i Breivika, Postboks 6418, 9294 Tromsø, Norway; E-Mail: aso023@post.uit.no

**Keywords:** *Bacillus* sp. AEMREG7, MBF-UFH, flocculating activity, glycoprotein, thermostable

## Abstract

A bioflocculant named MBF-UFH produced by a *Bacillus* species isolated from sediment samples of Algoa Bay of the Eastern Cape Province of South Africa was characterized. The bacterial identification was through 16S rDNA sequencing; nucleotide sequences were deposited in GenBank as *Bacillus* sp. AEMREG7 with Accession Number KP659187. The production of the bioflocculant was observed to be closely associated with cell growth. The bioflocculant had the highest flocculating activity of 83.2% after 72 h of cultivation, and approximately 1.6 g of purified MBF-UFH was recovered from 1 L of fermentation broth. Its chemical analyses indicated that it is a glycoprotein composed of polysaccharide (76%) and protein (14%). Fourier transform infrared spectroscopy (FTIR) revealed that it consisted of hydroxyl, amide, carboxyl and methoxyl as the functional moieties. Scanning electron microscopy (SEM) revealed the amorphous structure of MBF-UFH and flocculated kaolin clay particles. The maximum flocculating activity of 92.6% against kaolin clay suspension was achieved at 0.3 mg/mL over pH ranges of 3–11 with the peak flocculating rate at pH 8 in the presence of MgCl_2_. The bioflocculant retained high flocculating activity of 90% after heating at 100 °C for 1 h. MBF-UFH appears to have immense potential as an alternative to conventional chemical flocculants.

## 1. Introduction

The flocculation stage is a major phase in water treatment technology for the exclusion of both organic and inorganic pollutants [1]. Flocculants are substances used in the clumping of colloids, cells and suspended solids into larger sizeable flocs that can be removed effectively from solution by sedimentation [2]. Applications of flocculants have included downstream processes in the fermentation industries, as well as drinking and waste-water treatment facilities [3]. Flocculants may be categorized into three groups: organic flocculants, such as polyacrylamide derivatives; inorganic flocculants, such as polyaluminum chloride and ferric chloride; naturally-occurring flocculants, such as chitosan, sodium alginate and bioflocculants. However, the choice of flocculants has a major influence on the performance of the flocculation process [4].

Bioflocculants stands out among others, as they have the advantages of innocuousness, biocompatibility, biodegradability and environmentally friendliness, unlike organic and inorganic flocculants, which are toxic and whose degradation intermediates are difficult to remove from the environment [5]. Besides, organic flocculants, such as polyacrylamide and polyethylene imine derivatives, have been implicated in adverse human health effects [6]. An outstanding example is aluminum salts, which have been demonstrated to cause Alzheimer’s disease in humans [7]. Conversely, the enormous advantages associated with bioflocculants motivate its consideration as an alternative, hence the vast interest in the scientific and industrial community worldwide [8].

Bioflocculants are mostly composed of macromolecular substances, such as polysaccharides, protein, lipids and nucleic acids [9]. The chemical composition and flocculating efficiency of bioflocculants depends on some factors, including the nature of the environment in which bioflocculant-producing microorganisms are isolated, the media compositions in which the microorganisms are cultivated, the functional groups and molecular weight of the bioflocculant [10]. Several studies have demonstrated the efficiencies of bioflocculants in the treatment of drinking/waste waters and other downstream processing [6].

Nevertheless, low flocculating efficiency, low yields and high cost of production compared with the conventional flocculants are major limitations to large-scale production and application of bioflocculants [11,12,13]. Consequently, there is a need for continual exploration for novel microbes with efficient bioflocculant-producing capability. Thus, efficient bioflocculant-producing microbes should possess bioflocculants with high flocculating efficiency and yields [14,15].

Marine environment have remained a potential reservoir of microbes with novel metabolites, and the exploitation of this ecosystem through prospecting for microbes with novel metabolites is still very nascent [16,17,18,19,20]. Consequently, the current study aimed at characterizing the bioflocculant produced by *Bacillus* sp. AEMREG7 isolated from a sediment sample of Algoa Bay in the Eastern Cape Province of South Africa.

## 2. Results and Discussion

### 2.1. Time Course of Bioflocculant Production

**Figure 1:**
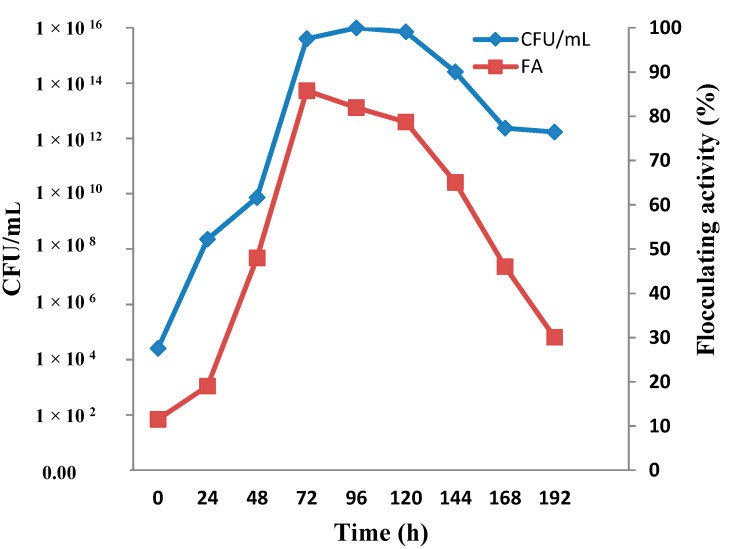
Time course of bioflocculant production by *Bacillus* sp. AEMREG7.

Figure 1 shows the relationship between MBF-UFH production and cell growth over a cultivation time of 192 h. Most microorganisms produced bioflocculant during the exponential growth phase [12]. The flocculating activity of the bioflocculant increased gradually with an increase in cultivation time. Generally, the production of MBF-UFH by *Bacillus* sp. AEMREG7 was observed to correspond with cell growth. Nonetheless, the highest flocculating activity of 83% was attained at 72 h in the late exponential growth phase, thus indicating that the production of MBF-UFH was closely associated with cell growth [21]. However, the decrease in the flocculating activity observed after 72 h may be due to the deflocculating enzyme released by the microorganism during the death phase of the cells. These results were similar to the findings of Wu and Ye [22] in which the bioflocculant production was synchronous with cell growth and reached maximum flocculating activity in the late logarithmic growth phase and early stationary phase. Furthermore, an increase in cultivation time led to a decrease in bioflocculant production as the flocculating activity decreased steadily. This decrease in flocculating activity of MBF-UFH might be due to deflocculating enzymatic activities or accumulation of toxic metabolic wastes affecting the produced bioflocculant [9]. Therefore, a cultivation time of 72 h was chosen for the subsequent experiments. It is apparent that MBF-UFH biosynthesis occurred during different microbial growth phases for different organisms [23]. Additionally, under optimum culture conditions, about 1.6 g of purified MBF-UFH was recovered from 1 L of fermentation broth of *Bacillus* sp. AEMREG7, which is higher than 0.4 g of the purified bioflocculant produced by *Aspergillus flavus* [24]. On the contrary, the bioflocculant production by *Chryseobacterium daeguense* W6 was released into the culture broth in the death phase of cell growth, when the nutrient in the medium had been depleted [10].

### 2.2. Fourier Transform Infrared Analysis

**Figure 2:**
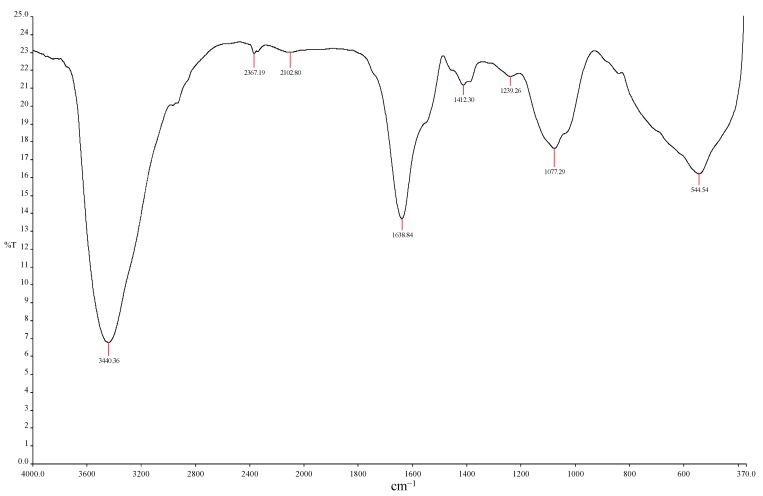
Fourier transform infrared (FTIR) spectra of purified MBF-UFH produced by *Bacillus* sp. AEMREG7.

The functional moieties in the molecular chain of MBF-UFH were identified with FTIR spectrophotometry (Figure 2). The spectrum displaced an intense broad stretching peak at 3440 cm^−1^, which indicated the presence of a hydroxyl or amide group [25,26]. The water solubility of the bioflocculant was attributed to the presence of hydroxyl group forming a hydrogen bond with a water molecule. A weak peak observed at 2367 cm^−1^ was either CO_2_ adsorption or may be from the amine group [27]. Furthermore, an asymmetric stretching peak was at 1638 cm^−1^, which showed the presence of carbonyl group stretching vibration in the peptide [28]. The peak detected at 1412 cm^−1^ could be ascribed to the symmetric stretching of the –COO^−^ group [18]. The presence of carboxyl groups provides more adsorption sites for particle attachment, so many particles can be adsorbed to the long molecular chain of the bioflocculant [29]. The absorption peaks ranging from 1000–1200 cm^−1^ were designated to C–O–C and C–O, which indicated the presence of polysaccharides [30,31]. The peak at 1077 cm^−1^ indicated the presence of methoxyl groups [9]. The characteristics of the FTIR spectrum showed the presence of hydroxyl, amide, carboxyl and methoxyl groups in the MBF-UFH structure as the main functional moieties preferred for flocculation [32].

### 2.3. Scanning Electron Microscopy Imaging

**Figure 3:**
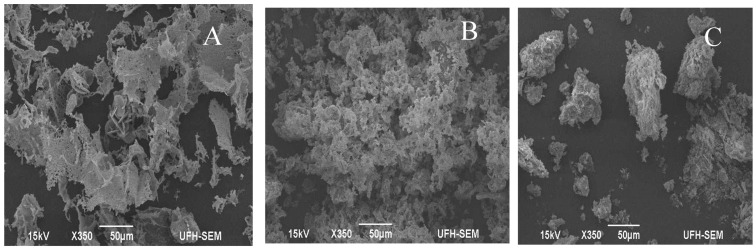
SEM imaging of purified MBF-UFH produced by *Bacillus* sp. AEMREG7 (**A**), kaolin clay particles (**B**) and kaolin clay suspension flocculated with MBF-UFH (**C**).

Surface morphology structure of the bioflocculant was explicated by scanning electron microscopy (SEM). SEM is a type of electron microscope that divulges the image of a sample by scanning it with a high-energy beam of electrons in a raster scan pattern [25]. The electrons interrelate with the atoms that make up the sample, producing signals that contain information about the sample’s surface structure [33]. The SEM image showed that MBF-UFH has an amorphous structure of a compact nature (Figure 3A). The configuration of this bioflocculant may be accountable for its high flocculation efficiency. Before the flocculation process, the kaolin clay particles appeared to be fine and scattered (Figure 3B), and after the flocculation process, the functional moieties in the molecular chain of MBF-UFH were used for attachment on the kaolin clay particle. Consequently, the interaction between the bioflocculant and kaolin clay particle resulted in the formation of flocs that later aggregated to larger sized flocs, which precipitated out of the suspension as the result of gravity (Figure 3C). This observation showed that bridging played a vital role in the flocculation process [34]. These results concur with previous findings for the purified bioflocculants produced by a consortium of *Streptomyces* and *Cellulomonas* species and *Nocardiopsis aegyptia* sp. nov. [6,17].

### 2.4. Elemental Analysis by Energy Dispersive X-ray Spectroscopy (EDX)

The EDX analysis of the purified MBF-UFH revealed its elemental composition in mass proportion (% *w*/*w*): C (17.21), N (6.66), O (40.04), Na (5.21), Mg (5.02), P (7.90), S (0.60), Cl (6.11), K (1.63) and Ca (9.63). Likewise, the presence of non-sugar components is usually small; nevertheless, they may provide flexibility and stabilize MBF-UFH [6]. In agreement with our observation, Singh *et al.* [35] documented that the exopolysaccharide produced by *Bacillus licheniformis* was composed of the following elements in mass proportion (% *w*/*w*): C (38.48), O (55.71), P (0.50), S (1.47), Cl (1.24), Ca (0.25), Na (2.34). Furthermore, Mabrouk [17] reported analogous cases with the bioflocculant produced by *Nocardiopsis aegyptia* sp. nov., which was composed of the following elements in mass proportion (% *w*/*w*): Na (7), P (6.9), S (1.9), Cl (66.3), K (16.9), Cu (0.5), Zn (0.6).

### 2.5. Physicochemical Properties of Purified Bioflocculant

#### 2.5.1. MBF-UFH Dosage

**Figure 4:**
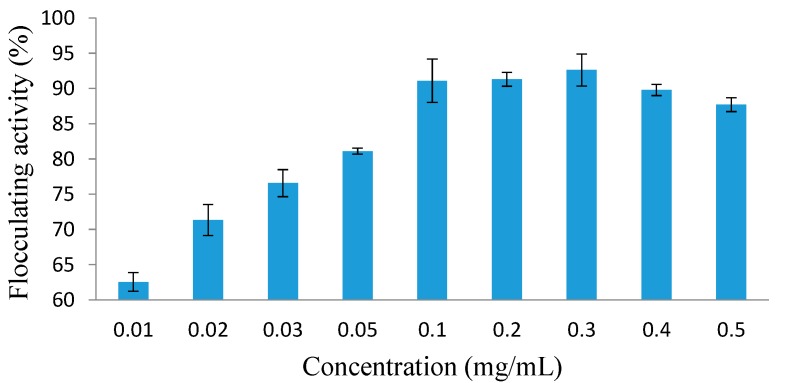
Effect of MBF-UFH concentration on the flocculating activity of purified bioflocculant produced by *Bacillus* sp. AEMREG7.

The effect of MBF-UFH dosage on flocculation efficiency for kaolin clay suspension was examined in an attempt to determine the most cost-effective dose for flocculation process. Under optimal conditions, the maximum flocculating activity is usually attained at the optimal bioflocculant dosage [36]. The flocculating activity of purified MBF-UFH was investigated in a range of 0.01–0.5 mg/mL (Figure 4). The flocculating activity of 61.5% was achieved at 0.01 mg/mL, and a further increase in MBF-UFH dosage resulted into a gradual decrease in flocculating activity. Nevertheless, the optimum bioflocculant dosage range for effective flocculation efficiency of over 90% was observed between 0.1 and 0.3 mg/mL, with the highest flocculating activity of 92.6% attained at 0.3 mg/mL. However, there was no significant increase in the flocculating activity of MBF-UFH when the dosage was increased from 0.1 up to 0.3 mg/mL (Figure 4). Although, the flocculating activity of MBF-UFH was low at 0.01 mg/mL (61.5%) compared to the flocculating activity noticed in the optimum range of 0.1–0.3 mg/mL (above 90%). At a lower dosage, MBF-UFH was relatively small to destabilize the negative charge of the kaolin clay particles, and the excess kaolin particles restabilized and increased the turbidity of the suspension; lower flocculating activity was noted in comparison to the flocculating rate observed at 0.1 mg/mL (Figure 4). This showed that the bridging effect of MBF-UFH was lower at 0.01 mg/mL compared to when it was at a higher dosage. On the contrary, the flocculating activity slightly decreased to 87.7% on increasing MBF-UFH dosage to 0.5 mg/mL compared with the flocculating activity of over 90% observed at an optimum dosage range between 0.1 and 0.3 mg/mL. This observation was in agreement with those reported elsewhere [37,38,39]. The decrease in flocculating activity of MBF-UFH observed at 0.5 mg/mL might be due to the over addition of the negatively-charged MBF-UFH, generating strong repulsive forces between the kaolin clay particles and the bioflocculant. These processes restabilized the suspended particles, increasing the viscosity of the suspension, blocking the adsorption sites and noticeably reduced floc formation [40]. These findings are consistence with previous studies reported by Elkady *et al.* [41] and Zheng *et al.* [9]. It has been extensively documented that a lower concentration of bioflocculants with a high flocculating efficiency will contribute to treatment cost reduction. Besides, information on the dosage requirement is substantial for future prospects in water treatment applications [28].

#### 2.5.2. Effect of Cations on the Flocculating Activity

In order to examine the effect of cations on the flocculating activity of MBF-UFH, a 4 g/L kaolin suspension in distilled water was used as the test material. Flocculation assays were set up in which CaCl_2_ was replaced by other cations when necessary, and the synergistic effects of cations were found in all of the cations investigated, except for Fe^3+^ (Table 1). It was observed that oppositely-charged cations reduced the particle surface charge density, so that the MBF-UFH molecules and kaolin particles could draw closer to each other, and the attractive forces were capable of overcoming the electrostatic repulsion to become effective. This interaction brought about the flocculation process of MBF-UFH. Factors influencing the effects of cations on flocculation vary; this may include surface charge capacity and the pH of the reaction mixture [3,37]. The studied bioflocculant alone flocculated kaolin clay suspension over 70% without the addition of cations. Prior to this study, very few bioflocculants have been reported to have a high flocculation rate without cations’ aid [39]. The synergistic effect of cations was observed most with monovalent and divalent cations, showing substantial enhancement on flocculating activity (with over 20%), with the highest flocculating rate observed with Mg^2+^ (Table 1). The flocculating activity of MBF-UFH was inhibited by Fe^3+^. However, a variation in flocculating activity was observed with Al^3+^, while Fe^3+^ may be accounted for by the surface charge availability of these cations in relation to the charge distributions of the bioflocculants. The trivalent cation could change the surface charge of kaolin particles and cover the adsorption sites, which led to a decrease in flocculating activity noticed in the presence of Al^3+^. Nonetheless, another possible scenario may be due to the effect of cation on the ionic strength of the suspension, which might also affect the conformations of MBF-UFH chain. Since MBF-UFH showed a flocculation rate at a lower dosage, it showed that bridging was substantially involved in the flocculation process. However, bridging flocculation requires that the MBF-UFH chain remains in an extended conformation; a higher charge density or higher proportion of charged groups favored the presence of the extended conformation of MBF-UFH chain, provided that all of the groups are either anionic or cationic by means of the electrostatic repulsion. Replacing divalent cations by trivalent cations increases notably the ionic strength of the suspension, and therefore, the electrostatic repulsive forces among charged groups of the bioflocculant chain decrease notably. This fact causes MBF-UFH chain conformation changes towards a more compact conformation, which is not able to form bridges. This might probably be the main cause of the poor flocculation ability of MBF-UFH in the presence of Al^3+^ and Fe^3+^. In addition, the synergistic effect of Al^3+^ with MBF-UFH and the antagonistic effect of Fe^3+^ with MBF-UFH thus lead to high flocculating activity for the Al^3+^ and lower flocculating activity in the presence of Fe^3+^ when the synergistic effects of trivalent cations on the flocculating activity of MBF-UFH were compared. There have been several reports on divalent cations stimulating the flocculating activity of bioflocculants [8,22]. Ostensibly, it seems that the monovalent and divalent cations help to neutralize negative charges on MBF-UFH and the suspended kaolin particles, shortening the distance between them, increasing the initial adsorption of MBF-UFH onto the kaolin particle and thus leading to floc formation and sedimentation [22,42]. It has been well documented that the addition of cations to suspensions is necessary to induce the effective flocculation capability of a bioflocculant; the effects of cations on the flocculating activity of MBF-UFH are similar to the previous studies by Okaiyeto *et al.* [43] on the bioflocculant produced by *Micrococcus* sp. Okoh. On the other hand, the bioflocculants produced by *Aspergillus flavus* and *Klebsiella pneumonia* were cation independent, which showed an outstanding performance in kaolin clay suspension without the addition of metal ions [24,39].

**Table 1:** Effect of cations on the flocculating activity of purified MBF-UFH

Cation	Na^+^	K^+^	Li^+^	Mg^2+^	Ca^2+^	Mn^2+^	Fe^3+^	Al^3+^	BA
FA	93.29	92.18	92.46	94.77	91.13	92.68	9.90	68.36	70.94
(%) ±SD	1.47	1.49	1.54	0.71	2.35	1.12	5.14	2.51	3.12

FA, flocculating activity; BA-Bioflocculant (MBF-UFH) alone.

**Figure 5:**
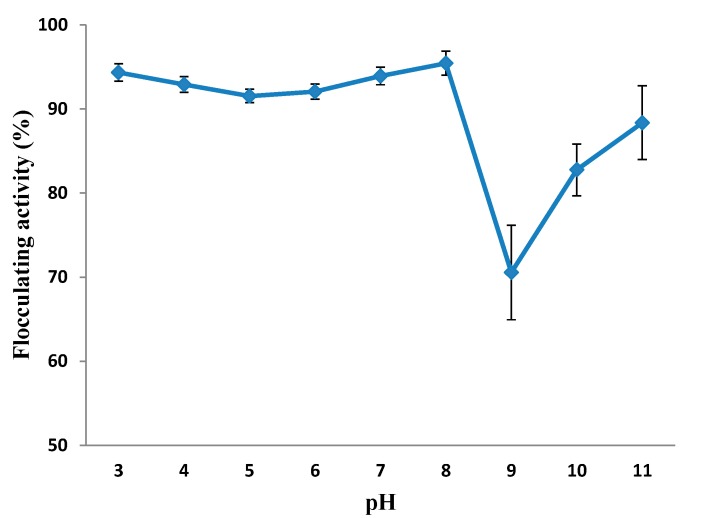
Effect of pH on the flocculating activity of purified MBF-UFH produced by *Bacillus* sp. AEMREG7.

#### 2.5.3. Effect of pH on the Flocculating Activity of Purified MBF-UFH

The pH of reaction mixtures is a key factor influencing the flocculation process [38]. One of the ways in which pH influences flocculating activity is by affecting the stability of suspended particles and floc formation [44]; the effect of pH on the flocculating activity of purified MBF-UFH was evaluated, and the results are depicted in Figure 5. The bioflocculant had a strong flocculating activity over a wide range of pH, 3–11. There was no significant difference in the flocculating activity of MBF-UFH between the pH ranges 3–8. However, the peak flocculating activity of 95.4% was achieved at weak alkaline condition, pH 8, and a sharp decline in flocculating activity was noticed at pH 9, which might be due to the fact that the surface charge spatial arrangement is both pH and temperature dependent. Thus, it would be safe to assume that the spatial charge arrangements for flocculation were not ambient at pH 9 and within the alkaline range. MBF-UFH was tolerant to the extreme pH and showed excellent flocculating activity in a strong acidic than basic condition. Hence, this suggested that MBF-UFH could be applied under acidic, neutral and alkaline circumstances, as it shows different electric states at different pH, which, in turn, influences the bridging efficiency of MBF-UFH for kaolin clay particles [38,45]. These observations are similar to the findings reported by Ugbenyen *et al*. [44] in which the flocculating activity of the bioflocculant produced by a consortium of *Cobetia* and *Bacillus* species was over 70% across a wide pH range of 3–11 with the highest flocculating activity attained at pH 8. The flocculating activity of the bioflocculant produced by *Bacillus* sp. F19 reached the maximum at pH 2 and maintained excellent flocculating activity within a range of pH 2–9. On the other hand, the bioflocculant produced by *Ruditapes* *philippinarum* flocculated over a wide pH range with the optimum flocculation rate between pH 7 and 9 [45].

#### 2.5.4. Thermal Stability of Purified MBF-UFH

**Figure 6:**
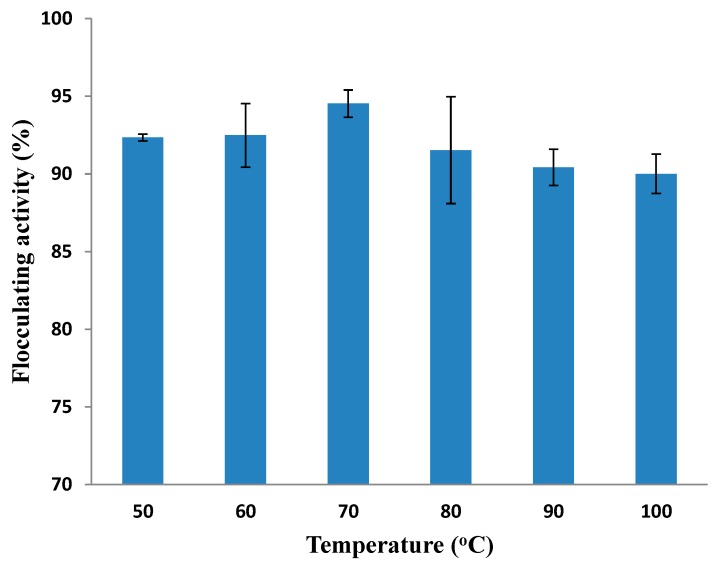
Thermal stability of purified MBF-UFH produced by *Bacillus* sp. AEMREG7.

The thermal stability of the purified bioflocculant was examined at 50, 60, 70, 80, 90 and 100 °C for 1 h at each temperature. As depicted in Figure 6, MBF-UFH had an exceptional flocculating activity of 90% over all of the temperature regimes. The high flocculating efficiency showed by MBF-UFH at 100 °C indicated that it is thermally stable and consisted predominantly of polysaccharide [46,47]. The thermal stability may be due to the presence of a hydroxyl group involved in the formation of hydrogen bonds in MBF-UFH structure [44]. The stability of MBF-UFH was similar to the bioflocculant produced by *C*. *glutamicum*, which retained high flocculating activity of 96.9% at 80 °C, but the stability decreased slightly on increasing the temperature to 100 °C [47]. Besides, the bioflocculant produced by *Aspergillus flavus* was thermally stable, which retained high flocculating activity above 90% over a temperature range of 10–100 °C [24]. Nevertheless, Salehizadel *et al.* [48] reported a less stable bioflocculant that lost about 50% of the flocculating activity after heating for 15 min at 100 °C.

### 2.6. Comparison of Flocculating Activity of MBF-UFH with Conventional Flocculants

The flocculating efficiency of MBF-UFH for kaolin clay suspension was compared with the conventional flocculants, such as aluminum chloride, ferric chloride and anionic polyacrylamide (Table 2). The maximum flocculation rate was achieved at the optimum concentration of these flocculants; and it was observed that polyacrylamide has the highest flocculating efficiency (94.30%), followed by MBF-UFH (91.1%), aluminum chloride (67.99%) and ferric chloride (42.78%). These findings showed that MBF-UFH was significantly more efficient than both aluminum chloride and FeCl_3_, but slightly less efficient than polyacrylamide. The results suggest the great potential of MBF-UFH as an alternative to the commonly-used flocculants in waste/drinking water treatment.

**Table 2:** Comparison of flocculating activity of MBF-UFH with conventional flocculants.

Flocculant	Conc. (mg/mL)	FA (%)
Aluminum chloride	1	67.99
FeCl_3_	1	42.78
Polyacrylamide	0.1	94.30
Bioflocculant	0.1	91.1

Conc., concentration.

### 2.7. Proposed Flocculation Mechanism of MBF-UFH

The presence of a hydroxyl group in the molecular chain of MBF-UFH suggested that ionic bonds might be responsible for the interaction between MBF-UFH molecules and the kaolin clay particle, which led to the high flocculating efficiency observed in our study [33]. The force of adsorption may have likely come from hydrogen bonds that were formed between OH groups of MBF-UFH and the kaolin clay particles. Besides, the presence of the hydroxyl group might also be responsible for the high solubility property of the bioflocculant. In addition, its thermal stability could be linked to the presence of hydroxyl group involved in the formation of hydrogen bonds in the MBF-UFH structure [44]. Likewise, the presence of carboxyl groups in MBF-UFH indicated that there were chemical bonds existing between the COO^−^ groups and kaolin clay particles. The carboxyl groups in MBF-UFH molecules were used as binding sites for the kaolin particle mediated by Mg^2+^. Therefore, we proposed that the flocculation process might be by bridging and charge neutralization.

Bridging was proposed to be involved in the ﬂocculation of MBF-UFH, since a high flocculating activity of 62.5% was observed even at a lower dosage of 0.01 mg/mL. Bridging occurred after the cation-kaolin complexes adsorbed onto the bioflocculant chains, leading to the formation of three-dimensional flocs, which were capable of rapid setting [22]. The MBF-UFH molecule extends the functional moieties beyond the particle’s surface to the solution and attracts other particles in solution, thereby resulting in the aggregation of bigger flocs that can easily precipitate out of solution as a result of the bridging effect, and this phenomenon was confirmed by the SEM observations.

The role of cations is to increase the adsorption of MBF-UFH molecules on the surface of suspended particles by decreasing the negative charge on both MBF-UFH and the kaolin particle. When Mg^2+^ was added to the kaolin suspension, a decrease in charge density occurs, which eventually reversed the negatively-charged particles to positive and, hence, led to inter-particle bridging between kaolin particles and MBF-UFH. The divalent cation Mg^2+^ weakened the electrostatic repulsive forces between the bioflocculant and the kaolin clay particles; shortening the distance between them by compressing the double layer of kaolin particles and increasing the initial adsorption of MBF-UFH on the kaolin clay particles. Thus, electrostatic happened to be the main acting force between bioflocculant and the kaolin particles. Consequently, the functional moieties in the MBF-UFH molecule (hydroxyl, carboxyl and amine) adsorbed the kaolin clay particles and formed hydrogen bonds with them. Thus, the negatively-charged carboxyl group (COO^−^) of MBF-UFH could react with the positively-charged site of the suspended kaolin particles. It can be assumed that cations stimulate ﬂocculation by neutralization and stabilization of residual negative charges of the carboxyl group of the bioflocculant MBF-UFH forming a bridge that binds kaolin particles to each other [22].

Moreover, since the chemical composition analyses revealed that MBF-UFH is composed of both polysaccharide and protein, this showed that the flocculation process might involve multiple functional moieties from both polysaccharide and protein. Multiple functional moieties imply many adsorption sites for the kaolin particles, which led to the high flocculating efficiency observed with MBF-UFH [49]. Furthermore, it was observed that MBF-UFH alone without cation flocculated kaolin clay suspension above 70%; this showed that the flocculation process could also be through charge neutralization as a result of electrostatic attraction between the positively-charged amino group of the protein part of MBF-UFH and the negative charge on the kaolin clay particle [50].

## 3. Experimental Section

### 3.1. Microorganism and Culture Conditions

*Bacillus* sp. AEMREG7 was previously isolated from a sediment sample of Algoa Bay in the Eastern Cape Province of South Africa, identified by 16S rDNA sequencing and preserved in 20% glycerol stock at −80 °C as part of the culture collection of the Applied and Environmental Microbiology Research Group (AEMREG), University of Fort Hare, South Africa. The activation medium was composed of 10 g tryptone, 3 g beef extract and 5 g NaCl in 1 L of filtered seawater [51]. The medium was sterile by autoclaving at 121 °C for 30 min and allowed to cool down at room temperature. The bacteria was activated by inoculating 5 µL of the glycerol stock into 5 mL of the activation medium and incubated overnight at 28 °C on a rotary shaker at 160 rpm. The growth medium was composed of 20 g glucose, 5 g K_2_HPO_4_, 2 g KH_2_PO_4_, 0.3 g (NH_4_)_2_SO_4_, 0.5 g urea, 0.5 g yeast extract, 0.3 g MgSO_4_·7H_2_O, 0.1 g NaCl in 1 L of filtered seawater [52]. After 24 h of fermentation, 2 mL of the fermented broth were inoculated into 50 mL of sterile growth medium incubated on rotary shaker at 160 rpm, 28 °C for 72 h. The flocculating activity of the cell-free supernatant was determined by flocculation assay after separating the bacterial cells from the culture broth by centrifugation at 4000 rpm for 30 min.

### 3.2. Determination of Flocculating Activity

A kaolin clay suspension was used as a model for the natural turbidity of raw surface water to determine the flocculating activity of the bioflocculant produced by *Bacillus* sp. AEMREG7. Four grams of kaolin clay (Merck, Darmstadt, Germany) were suspended in 1 L of distilled water to make the kaolin suspension. Two milliliters of cell-free supernatant were added to 100 mL of kaolin suspension in 250-mL conical flask containing 3 mL of CaCl_2_ (1% *w*/*v*). The mixture was agitated for 60 s, transferred to a 100-mL gradual measuring cylinder and allowed to stand for 5 min. The optical density (OD) of the supernatant was measured using a spectrophotometer (Helios Epsilon, USA) at 550 nm. The control experiment was set up by replacing the bioflocculant with 2 mL of freshly-prepared un-inoculated medium. The flocculating efficiency was calculated with the following equation:

*Flocculating Efficiency* (%) *=* (*A* − *B/A*) × 100%

where *A* and *B* are optical densities of the control and sample read at 550 nm, respectively.

### 3.3. Time Course of MBF-UFH Production

The time course of MBF-UFH production was done according the method described by Liu *et al.* [53] and Nwodo *et al.* [18] with some modifications. The culture broth (24 h old) was adjusted to OD_660_ 0.1; then, the standardized culture broth was inoculated into 200 mL of fermentation medium contained in 500 mL flasks. The flasks were placed in a rotary shaker at 160 rpm, 28 °C for 192 h. The production of MBF-UFH was monitored over time by withdrawing 10 mL of the culture broth at intervals of 24 h for flocculating activity determination and bacterial cell growth via viable cell count (CFU/mL).

### 3.4. Bioflocculant Purification

The purification of MBF-UFH was carried out according to the description of Cosa *et al.* [54] and Li *et al.* [55] and with some modifications. After 72 h of fermentation, the culture broth was centrifuged at 4000 rpm for 30 min at 15 °C. The viscosity of the culture broth was reduced by diluting with two volumes of distilled water and then centrifuged at 4000 rpm for 30 min in order to remove bacterial cells. The pellet was discarded, and two volumes of cold ethanol were added to the supernatant and kept at 4 °C overnight for ethanol precipitation. The resulting precipitate was collected by centrifugation at 4000 rpm for 30 min at 15 °C and lyophilized to obtain crude MBF-UFH. The crude bioflocculant was dissolved in 100 mL of distilled water, to which one volume of a mixture of chloroform and *n*-butyl alcohol (5:2 *v*/*v*) was added, stirred for 60 s and kept overnight at room temperature. The upper phase was collected by centrifugation at 4000 rpm for 30 min at 15 °C, vacuum-dried and re-dissolved in distilled water. MBF-UFH solution was dialyzed against de-ionized water overnight and then lyophilized to obtain a purified MBF-UFH.

### 3.5. Chemical Composition Analysis of a Purified MBF-UFH

The total sugar content of MBF-UFH was determined according to the phenol-sulfuric method described by Chaplin and Kennedy [56] using glucose as the standard. The total protein contain was determined by the Bradford method described by Bradford [57] using bovine serum albumin (BSA) as the standard. The functional moieties in the MBF-UFH molecule were identified using an FTIR analyzer (Perkin Elmer System 2000, Buckinghamshire, UK). Dried MBF-UFH sample was ground with KBr powder and pressed into pellets for FTIR spectra measurement in the frequency range of 4000−400 cm^−1^ [33]. The surface morphology of MBF-UFH and kaolin clay particles was observed and elucidated using scanning electron microscopy (SEM) JEOL (JSM-6390LV, Tokyo, Japan). Five milligrams of MBF-UFH, kaolin clay and dried flocculated kaolin suspension were added on slides separately and fixed by air-drying. The fixed specimens were coated with gold and examined under SEM [58]. The elemental analysis was carried out with SEM equipped with Noran Six 200 Energy Dispersive X-ray (JEOL Ltd., Tokyo, Japan) [26].

### 3.6. Physicochemical Properties of a Partially-Purified Bioflocculant

#### 3.6.1. MBF-UFH Concentration

Different concentrations (0.05, 0.1, 0.2, 0.3, 0.4 and 0.5 mg/mL) of purified MBF-UFH were made with distilled water, and the flocculating activity of each was determined by the flocculation assay [37].

#### 3.6.2. Effect of Cations on Flocculating Activity

The synergistic effect of cations on the flocculating activity of purified MBF-UFH was investigated in accordance with the description of Zhao *et al.* [39]. The cation candidates included the metal of the following salts: NaCl, KCl, LiCl, MgCl_2_, CaCl_2_, MnCl_2_, FeCl_3_ and AlCl_3_. The flocculation assays were determined as described above, but CaCl_2_ was replaced by the solution of the aforementioned salts.

#### 3.6.3. Effect of pH on the Flocculating Activity of MBF-UFH

The effect of pH on the flocculating activity was evaluated at a pH value ranging from 3–11; 4 g/L of the kaolin clay suspension was made and divided into 9 groups, and the pH of each group was adjusted accordingly with 0.1 M HCl and 0.1 M NaOH. The flocculating activity was determined while other conditions were kept constant [59].

#### 3.6.4. Thermal Stability of MBF-UFH

MBF-UFH was dissolved in 60 mL of distilled water; 10 mL each of the bioflocculant solution were heated at different temperatures 50, 60, 70, 80, 90 and 100 °C for 1 h [50]. The bioflocculant solutions were allowed to cool, and their resulting flocculating activities were measured at room temperature, as described elsewhere [31].

### 3.7. Comparison of Flocculating Efficiencies of MBF-UFH and Conventional Flocculants

The flocculating efficiency of MBF-UFH and the conventionally-used chemical flocculants were compared by the flocculation assay in accordance with the description of Ugbenyen *et al.* [34] with some modifications. The chemical flocculants included: Alum, ferric chloride, anionic polyacrylamide (BDH chemicals Ltd.; Poole, UK, molecular weight ≥ 5000 KDa). Each of the flocculants was prepared by dissolving an appropriate concentration in distilled water, and jar tests were employed as described by Wang *et al.* [60]; the flocculating activities were measured separately afterwards.

### 3.8. Statistical Analysis

All data were treated in replicates, and the mean values were taken. Data were subjected to one-way analysis of variance (ANOVA) using the MINITAB Student Release 12 statistical package. A significance level of *p* ˂ 0.05 was used.

## 4. Conclusions

Bioflocculants are proposed to possess enormous industrial and biotechnological significance consequent to the advantages associated with them over conventionally-used flocculants. MBF-UFH produced by *Bacillus* sp. AEMREG7 under optimum fermentation conditions of 28 °C, 160 rpm and pH of six showed high flocculation activity of 83.2%. Bioflocculant production was, likewise, observed to be closely associated with cell growth. MBF-UFH’s composition was predominantly polysaccharides and proteins. The FTIR spectrum indicated the presence of hydroxyl and carboxyl in its molecular chain, which is important for flocculation. The SEM imaging illustrated the morphology and structural arrangements of MBF-UFH. As compared to conventionally-used flocculants, MBF-UFH demonstrated a high flocculating activity at a low dosage; a characteristic feature that demonstrates its suitability for industrial application. Thus, MBF-UFH potentially stands as an alternative candidate to chemical flocculants, hence making it attractive for further research and possible development for industrial-scale application.
